# Use of metabolomics for the identification and validation of clinical biomarkers for preterm birth: Preterm SAMBA

**DOI:** 10.1186/s12884-016-1006-9

**Published:** 2016-08-08

**Authors:** Jose G. Cecatti, Renato T. Souza, Karolina Sulek, Maria L. Costa, Louise C. Kenny, Lesley M. McCowan, Rodolfo C. Pacagnella, Silas G. Villas-Boas, Jussara Mayrink, Renato Passini, Kleber G. Franchini, Philip N. Baker, for the Preterm SAMBA and SCOPE study groups, Mary A. Parpinelli, Jussara Mayrink, Kleber G. Franchini, Iracema M. Calderon, Bianca F. Cassettari, Janete Vetorazzi, Lucia Pfitscher, Edilberto P. Rocha Filho, Débora F. Leite, Francisco E. Feitosa, Carolina L. Costa e Silva, Lucilla Poston, Jenny E. Myers, Nigel A.B. Simpson, James J. Walker, Gus A. Dekker, Claire T. Roberts

**Affiliations:** 1 Department of Obstetrics and Gynecology, University of Campinas (UNICAMP) School of Medical Sciences, R. Alexander Fleming, 101, Campinas, SP, 13083-881 Brazil; 2 Gravida: National Centre for Growth & Development, Liggins Institute, University of Auckland, Auckland, New Zealand; 3 Irish Centre for Fetal and Neonatal Translational Research (INFANT), Department of Obstetrics and Gynaecology, University College Cork, Cork, Ireland; 4 South Auckland Clinical School, Faculty of Medical and Health Sciences, University of Auckland, Auckland, New Zealand; 5 School of Biological Sciences, University of Auckland, Auckland, New Zealand; 6 LNBio–Brazilian Biosciences National Laboratory and School of Medical Sciences, University of Campinas (UNICAMP), Campinas, SP Brazil

**Keywords:** Spontaneous preterm birth, Metabolomics, Prediction, Biological biomarker, Mass spectrometry

## Abstract

**Background:**

Spontaneous preterm birth is a complex syndrome with multiple pathways interactions determining its occurrence, including genetic, immunological, physiologic, biochemical and environmental factors. Despite great worldwide efforts in preterm birth prevention, there are no recent effective therapeutic strategies able to decrease spontaneous preterm birth rates or their consequent neonatal morbidity/mortality. The Preterm SAMBA study will associate metabolomics technologies to identify clinical and metabolite predictors for preterm birth. These innovative and unbiased techniques might be a strategic key to advance spontaneous preterm birth prediction.

**Methods/design:**

Preterm SAMBA study consists of a discovery phase to identify biophysical and untargeted metabolomics from blood and hair samples associated with preterm birth, plus a validation phase to evaluate the performance of the predictive modelling. The first phase, a case–control study, will randomly select 100 women who had a spontaneous preterm birth (before 37 weeks) and 100 women who had term birth in the Cork Ireland and Auckland New Zealand cohorts within the SCOPE study, an international consortium aimed to identify potential metabolomic predictors using biophysical data and blood samples collected at 20 weeks of gestation. The validation phase will recruit 1150 Brazilian pregnant women from five participant centres and will collect blood and hair samples at 20 weeks of gestation to evaluate the performance of the algorithm model (sensitivity, specificity, predictive values and likelihood ratios) in predicting spontaneous preterm birth (before 34 weeks, with a secondary analysis of delivery before 37 weeks).

**Discussion:**

The Preterm SAMBA study intends to step forward on preterm birth prediction using metabolomics techniques, and accurate protocols for sample collection among multi-ethnic populations. The use of metabolomics in medical science research is innovative and promises to provide solutions for disorders with multiple complex underlying determinants such as spontaneous preterm birth.

## Background

Despite improvements in antenatal and neonatal care, the number of premature newborns each year has not significantly decreased since the 1960s. Preterm birth (PTB) is the leading cause of neonatal morbidity and mortality and a major contributor to loss of life, long-term disability, and health care costs [1–4]. The associated morbidity, mortality and high health costs have been well documented with premature infants facing life-threatening short and long term complications [5–8].

Despite the enormity of the health economic burden of preterm birth, and many years of focused research, a common aetiology and/or predictive test have not yet been identified. Spontaneous preterm birth (sPTB) is considered one of the “Great Obstetrical Syndromes”, which are conditions resulting from complex interactions between the maternal and fetal genome and the environment and which have a long preclinical period, fetal involvement and adaptive functioning in nature [9]. This reflects the multifactorial nature of this condition and the need to apply strategies that are capable of identifying multiple markers simultaneously in parallel with the assessment of clinical and biophysical risk factors.

There are many clinical [10–13] and biochemical risk factors [14–16] associated with sPTB and it is likely that these biochemical markers are present in the maternal blood long before the onset of a preterm labour. However, although certain candidate-driven approaches to studying these changes show promise, this has not resulted in effective predictive biomarkers for the general pregnant population. Due to these complex and dynamic characteristics of sPTB syndrome, it remains a difficult task to identify women and babies at risk.

Currently, the selection of women likely to deliver prematurely from clinical risk factors alone lacks the sensitivity required to effectively identify the majority of patients at risk of idiopathic sPTB [14]. Furthermore, parameters derived from previous obstetric history cannot be applied to nulliparous women. The association of biophysical predictors such as cervical length and/or vaginal biomarkers (fibronectin and phosphorylated insulin-like growth factor binding protein-1) enhances accuracy for prediction and enables more effective interventions for selected women. There are therapeutic interventions available for the prevention of sPTB, such as the use of progesterone [10, 17, 18] and cervical pessary [19]. Despite advances in selection of eligible women for such therapeutic interventions, the efficacy of cervical length or fetal fibronectin levels in asymptomatic women are still limited and seem to be more capable of discriminating women at lower risk than those at higher risk [20–22]. Owen et al. showed that almost 50 % of women with cervical length between 15 and 25 mm did not deliver before 35 weeks, as well as approximately 70 % with cervical length between 25–30 mm [23].

The development of a predictive test for spontaneous preterm birth would help to accurately identify a high-risk population. To be effective, therapies need to be commenced at a gestational age in which they are likely to be of benefit. A sensitive early pregnancy-screening test would facilitate the timely administration of prophylactic treatments to those women at highest risk. The development of physics, biology and medicine translational research can provide a comprehensive approach for biological processes with complex pathways and regulations. Metabolomics offers an unbiased hypothesis generating approach to identify and validate potential candidate metabolomic biomarkers [24, 25].

We propose a multi-strategy approach to biomarker discovery and validation through the establishment of a large early pregnancy biobank of appropriate samples, in conjunction with the application of analytical methods capable of quantifying multiple blood-borne species simultaneously, and using some clinical and epidemiological markers to identify women at highest risk of spontaneous preterm birth.

The development of predictive tests that translate into clinical care can be divided into two distinct phases; (i) hypothesis generation after acquisition of data, a non-biased process where no or limited biological knowledge is required and (ii) validation of generated hypotheses [26]. The Preterm SAMBA study goal spans both phases and aims to identify a clinically useful early pregnancy-screening test to ascertain which pregnancies are at risk of developing sPTB. Discovery-based methods will be applied to blood and hair samples taken from carefully matched phenotypes in both cohorts (preterm and term deliveries) to develop a predictive algorithm to identify those women at increased risk of sPTB and test the effectiveness of such an algorithm in a prospective cohort.

## Methods/design

Preterm SAMBA, an international collaborative multicentre study for the development of predictive tests that translate into clinical care, can be divided into two distinct phases: The first component (Discovery phase) is a case–control study that aims to identify clinical and metabolomics biomarkers related to spontaneous preterm birth. For this initial phase, untargeted metabolomics techniques will be employed to identify and quantify potential predictor’s metabolites that can be associated to potential clinical predictors. The second component (Validation phase) is a cohort study developed to validate the algorithm of prediction using the clinical and metabolomics biomarkers discovered in the first component of the study. Thus, to evaluate the performance of the prediction model developed at the first phase, targeted metabolomics techniques will be employed to analyse participants’ blood and hair samples to quantify those specific metabolites identified as potential predictors of preterm birth.

### Discovery phase

The initial phase of the project consists of a case–control study utilizing data and samples collected for the SCOPE study (Screening for Pregnancy Endpoints study). The SCOPE consortium was an international effort to determine the causes and potential predictors for pregnancy complications and its methodology had already been previously published [27–29]. Briefly, the cohort comprised 5690 healthy pregnant women recruited between November 2004 and August 2008 in New Zealand, Australia, Ireland and United Kingdom. Inclusion and exclusion criteria for the SCOPE study are described in Tables 1 and 2, respectively. Exclusion criteria include major fetal anomaly, chronic hypertension, diabetes, renal disease, systemic lupus erythematous, and antiphospholipid syndrome. These will therefore be the same criteria for the current study.

**Table 1:** Inclusion criteria of Preterm SAMBA validation phase – Brazilian cohort

• Singleton pregnancy	
• Nulliparous (no previous delivery ≥20 weeks)	
• Up to 21 weeks of gestational age

**Table 2:** Exclusion criteria of Preterm SAMBA validation phase – Brazilian cohort

• Unsure LMP and unwilling to have dating US	• Major Uterine Anomaly
• ≥3 Miscarriages	• Cervical Suture
• Major Foetal Anomaly/Abnormal Karyotype	• Knife cone biopsy
• Essential Hypertension Treated Pre-pregnancy	• Ruptured membranes now
• Mod-Severe Hypertension at booking (≥160/100 mmHg)	• Long term Steroids
• Pre-pregnancy Diabetes	• Low-dose Aspirin
• Renal Disease	• Calcium (>1 g/24 h)
• Systemic Lupus Erythematosus	• Eicosapentaenoic acid (fish oil)
• Anti-phospholipid Syndrome	• Vit. C ≥1000 mg & Vit. E ≥400 UI
• Sickle Cell Disease	• Heparin/LMW Heparin
• HIV or Hep B or Hep C positive	

Extensive sociodemographic and physical data will be collected including age, ethnicity, socio-economic status, dietary and lifestyle questionnaire, parity, BMI (body mass index) and cigarette smoking.

Plasma and serum samples will be collected at 20 weeks of gestation using stringent standard operating procedures designed for metabolomics studies, barcoded and stored at −80 °C within 2–4 h; the timing between collection and freezing will be known for all specimens.

Several Standard Operating Procedures (SOP) for sample preparation by removal of proteins via ultrafiltration were developed and validated. The analysis of deproteinized plasma samples will be performed employing gas chromatography and liquid chromatography mass spectrometry (GC-MS and LC-MS). GC-MS and LC-MS techniques will be performed as described previously [30, 31]. Quality control samples (acquired by pooling plasma from all subjects) will be interspersed in every 5th run to assess reproducibility and validity. It is envisaged that the socioeconomic/physical/biomarker discovery phase of the Preterm SAMBA study will identify several candidate markers and predictive multivariate models. Discriminatory metabolites will be translated to a targeted triple quad MS (QQQ-MS) platform, to be used in the validation phase.

**Fig. 1:**
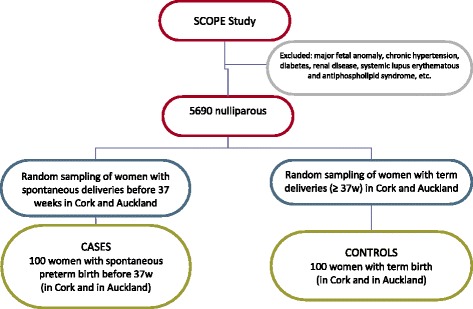
Flowchart of Preterm SAMBA Discovery Phase participants (Case-control component)

The proportion of pregnancies complicated by any preterm birth is approximately 10 %. Preterm SAMBA Discovery Phase will randomly select 100 women (*n* = 100) whose pregnancies reached term as compared to fifty randomly sampled pregnancies (*n* = 50) complicated by spontaneous preterm birth prior to 37 weeks gestation, in each of the Cork Ireland and Auckland New Zealand SCOPE cohorts (Fig. 1). The 20th week samples and data will be analysed to identify sPTB potential predictors. Considering there were no previous studies on this topic for preterm birth, these numbers were empirically estimated using a similar study performed for preeclampsia [31]. Using a type I error of 0.01, type II error of 0.10, a ratio between controls and cases of 1:1, an AUC of 0.9 and an OR of 10, the estimated sample of preterm birth is 49. We then anticipated around 50 preterm birth for each of the two centers.

### Validation phase – the Brazilian multicentre cohort study

The Preterm SAMBA validation phase consists of a Brazilian multicentre cohort study with 1150 low-risk pregnant nulliparous women. Five of the 27 members of the Brazilian Network for Studies on Reproductive and Perinatal Health (BNSRPH), were chosen to participate in the Brazilian cohort (Table 3). Previous excellence performance in epidemiological and translational studies and diversity of cultural, ethnical and sociodemographic population characteristics were criteria for centre selection. Therefore, there are participating centres in three of the five regions of Brazil, which are the three most populated regions of the country: Northeast, Southeast and South.

**Table 3:** Participating centres in the Preterm-SAMBA study validation phase – Brazilian cohort

Maternity of CAISM, University of Campinas, in Campinas, São Paulo.	
Maternity of the School of Medicine from UNESP, in Botucatu, São Paulo.	
Maternity of the Clinic Hospital, Federal University of Rio Grande do Sul, in Porto Alegre, Rio Grande do Sul.	
Maternity of the Clinic Hospital, Federal University of Pernambuco, in Recife, Pernambuco.	
MEAC – School Maternity of the Federal University of Ceará, in Fortaleza, Ceará.	

Assuming a type I error rate, α, of 5 % and an estimated area under ROC curve of at least 0.68, then in order to test hypotheses to a suitable level of power (80 % power, β = 0.2), the sample size sufficient should approximate to 80 cases of spontaneous preterm birth (<34 weeks gestation), calculated using MEDCALC®. Based on a minimum expected preterm birth rate of 7 %, the total cohort size should therefore be of approximately 1150 subjects, around 230 women at each participating centres.

#### Recruitment and data collection

The recruitment strategies include approaching existing pregnant women in participating facilities during prenatal care visits and with website/internet, flyers and local community advertisings. After the identification of potential participants, the research assistant will invite women and obtain an informed consent form of those who meet the inclusion criteria and agree to participate. Maternal age and ethnicity will be recorded from all approached women to facilitate a comparison of those who are recruited and those who decline.

All collected data will preferably be entered directly into the database, but printed forms will also be available in case of inability to access the internet-based database. In such cases, the data will be then entered later and completed printed forms will be stored, according to the required ethical principles.

#### Sociodemographic, physical data and pregnancy outcomes

*First Visit (19–21 weeks):* similarly to the SCOPE study, detailed information of sociodemographic characteristics (age, socioeconomic status, education, ethnicity, occupation and type of maternity care), maternal medical and obstetric history, infertility history, drugs and medications use, family medical and obstetric history and current pregnancy (occurrence and details of infection, vaginal bleeding, dipstick proteinuria, intercourse and hospital admission) will be collected.

Anthropometric measurements of maternal body mass index, height, weight, head circumference, arm circumference and triceps, biceps subscapular and suprailiac skinfolds will be performed according to standardized techniques. Height and weight of lightly clothed women will be measured to the nearest 0.1 mm and 0.1 kg respectively. Head and arm circumferences will be measured with an inelastic tape and skinfold thicknesses will be measured on the same side of the body to the nearest 0.2 mm using Harpenden (and/or Lange) skinfold calliper. The calliper is placed 1 cm distal to the firmly grasped skinfold, using the thumb and the index finger, at 90° to the skin. A single measurement is taken after 2 s.

Dietary intake will be assessed using a 24-h dietary recall administered by a trained professional who will query participants about food and beverage consumption in the previous 24 h. A trained nutritionist will then estimate calories, macro and micronutrient intake using computer-based standard tables allowing for appropriate ethnic, social and regional variations.

Furthermore, three consecutive manual blood pressure measurements will be recorded, using an appropriate cuff size for different arm circumferences and using Korotkoff phase V for diastolic blood pressure.

*Second and third Visit (27–29 weeks and 37–39 weeks; both optional):* three consecutive manual blood pressure measurements, anthropometric parameters (weight, height, head and arm circumference and triceps, biceps, subscapular and suprailiac skinfolds) and occurrence and characteristics of infection, vaginal bleeding, dipstick proteinuria, intercourse and hospital admissions will be recorded.

*Postpartum data:* data will be collected from the participant’s medical record, the prenatal chart and/or from a personal interview with the participant during hospital admission to minimize missing information. The main outcome is spontaneous preterm birth, defined as a birth before 34 weeks of gestational age due to preterm labour or premature rupture of membranes. Secondary outcomes will also be evaluated: spontaneous preterm birth alternatively defined as a birth before 37 weeks of gestational age due to preterm labour or premature rupture of membranes, provider-initiated preterm birth, defined as preterm birth due to medical indication on account of maternal or fetal conditions; pre-eclampsia, defined as having systolic blood pressure ≥ 140 or systolic blood pressure ≥ 90 mmHg after 20 weeks gestation on at least two occasions apart of 20 min, and/or proteinuria (24-h urinary protein ≥ 300 mg or urine dipstick ≥ ++) and/or severe maternal complications [32]; gestational diabetes mellitus according to ADA guidelines [33]; fetal growth restriction (FGR) defined as having birthweight below 10th percentile based on GROW customised birthweight centiles [34]. Clinical data will also be collected regarding the occurrence of preterm labour, cervical cerclage, deep vein thrombosis, infection, vaginal bleeding, dipstick proteinuria, intercourse, hospital admission, deep vein thrombosis during pregnancy and puerperium, and maternal mortality and the use of progesterone and/or pessary, tocolytic, antibiotic for preterm labour or pPROM, corticosteroids for fetal maturation, magnesium sulphate for neuroprotection during pregnancy. The occurrence of severe maternal morbidity and near miss will also be reported according to WHO guidelines [35]. Neonatal outcomes related to neonatal morbidity and mortality will be recorded until newborn discharge or death.

#### Sample collection, processing and storage

**Fig. 2:**
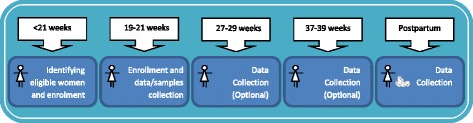
Visits of Preterm-SAMBA Validation Phase

Non-fasting blood samples will be collected at 20 (between 19 and 21) weeks of gestational age (Fig. 2). All research assistants will be trained according to specific and detailed Standard Operation Protocols (SOPs) developed for sample collection, processing and storage. One of the study coordinators was trained by the SCOPE team, to guarantee adequate understanding of all necessary procedures. A maximum of 20 mL of blood will be collected to provide serum and plasma specimens. The blood specimens will be stored in 250 μL 2-D barcoded cryovials after one centrifugation cycle for plasma specimens (2000×g for 10 min at 4 °C) and two centrifugation cycles for serum (2000×g for 10 min and 2400×g for 10 min at 4 °C). The blood samples will be processed and frozen at −80 °C within 2–4 h. The time interval between collection and freezing will be recorded for all specimens.

Although the Preterm SAMBA strategy and workflow is focussed around the analysis of blood samples, we recently reported a proof-of-concept study, which highlighted the potential use of the hair metabolome in the prediction of pregnancy complications [36]. Hair samples will also be collected at 20 weeks’ gestation. Samples (20–30 hair strands for each participant) will be collected from the occipital area, 0.5 cm away from the scalp, using blunt scissors. Then, hair will be packed in aluminium foil and stored at room temperature [36]. A unique linear barcode will be pasted on each hair package. All specimens and quality control information will be registered in the database.

#### Database

A specific database for the Preterm SAMBA Brazilian Cohort was developed together with MedSciNet, a Swedish based company specialized in the design and development of online database systems linked with biobanks management systems, similarly to the database previously used for the SCOPE. The preterm SAMBA database will be centralized, secure, internet-based and FDA (United States Food and Drug Administration) and HIPAA (Health Insurance and Accountability Act of 1996, United States Security and Privacy Rules) compliant, which allows continuous data entry and monitoring of study progress. Completeness of clinical data and specimen collection will be constantly monitored, with incomplete fields ‘flagged’ for attention. The database allows several monitoring procedures with hierarchical access licenses and tracking system for all specimen aliquots stored. To comply with biobank regulatory issues, patients will only be identified by a unique study number. Pseudo-anonymised metadata and interim data will be stored using our laboratory information management system. The identifying information about participants will be kept in a separate and secure local database.

#### Data and sample quality

Several procedures to enhance and assure data and sample quality will be adopted. All entered data will be prospectively and retrospectively monitored. During data entry, internal consistency of variables is performed and error messages are automatically flagged. After completing the collection of data from a participant, all information needs to be reviewed by a local monitor. Then, the final form has to be signed by the local principal investigator (PI) in order to be incorporated in the final database. The coordinating centre (Campinas, Brazil) will also perform a centralized monitoring of data and samples. An initial meeting with all researchers from Brazilian participating centres has been held to discuss the final protocol, procedures to be implemented, their particular characteristics and necessary approaches to be used to guarantee the implementation of the study. Another general meeting at the end of study is planned in order to discuss results, strategies for manuscripts’ writing and submission and other related topics.

The coordinating centre will randomly select approximately 10 % of printed completed forms to carry out a check and validation of data from the forms and database entry during the first and second half of the study. This double-check procedure enhances data quality and decreases typing errors.

The record of information regarding sample collection, processing (precentrifugation and centrifugation) and storage processes will follow the Standard Preanalytical Coding for biospecimens (SPRECs) protocol, developed and recommended by the International Society for Biological and Environmental Repositories Biospecimen (ISBER) Science Working Group [37]. This protocol enables standardization of preanalytical information, using standard codes to refer to the techniques and conditions to which the samples were submitted.

#### Metabolomics analysis

The precise methodology to be used in the validation phase will depend on the ongoing discovery studies. As detailed above we anticipate that it will be based on a targeted triple quad MS (QQQ-MS) platform, as previously described [38]. We will subsequently describe details relating to metabolomics analysis techniques and metabolomics statistical analyses. We anticipate that data analysis will be integrated into the relational database such that decision rules may combine both clinical and spectrometric data.

The performance of the final algorithm developed in the discovery phase will be evaluated by its capacity to predict spontaneous preterm birth occurrence in women from the Brazilian cohort. The validation will be performed using the average squared difference between predicted and observed outcome (R^2^), adjusted R^2^ (same as R^2^, but penalizes for the number of predictors), sensitivity, specificity, positive and negative predictive values, likelihood ratio and the area under the ROC curve.

### Ancillary studies

The Preterm SAMBA Brazilian cohort study will collect additional data regarding other relevant maternal and fetal obstetric complications. Detailed clinical data related to the occurrence and severity of pre-eclampsia, fetal growth restriction and gestational diabetes mellitus will be recorded. Fetal growth restriction will be diagnosed if birthweight is below 10th customised percentile. The occurrence of severe maternal morbidity, maternal near miss and maternal mortality during pregnancy or up to discharge after delivery will also be recorded, according to WHO definitions [35].

### Ethical aspects

The SCOPE study, whose data and samples will be analysed for the Preterm SAMBA discovery phase, was approved by local ethics committees in New Zealand and Ireland and registered in the Australian and New Zealand Clinical Trial Registry (ACTRN12607000551493) [28]. All women who participated in the SCOPE study provided written informed consent and agreed to have their data and samples used in other studies. The Preterm SAMBA study has been reviewed and approved by the National Committee for Ethics in Research of Brazil (CONEP) and by the Institutional Review Board (IRB) of the coordinating centre (Letter of approval 1.048.565 issued on 28^th^ April 2015) and of all other Brazilian participating centres. All women who will be enrolled in the Preterm SAMBA Brazilian cohort (Validation phase) will sign an informed consent form, also allowing for future additional studies with their biological samples without any additional consent.

The ethical principles stated in the Brazilian National Heath Council (Resolution CNS 466/12) will be respected in every stage of this study. The anonymity of the source of information will be guaranteed and the care for the women will be provided independent of her agreement to participate in the study. All ethical principles related to biobank storage and transport will be followed according to national and international rules related to research with human beings. The study also complies with the Declaration of Helsinki amended in Hong Kong in 1989. The methodological and ethical aspects of Preterm SAMBA study protocol were developed following STROBE guidelines [39].

## Discussion

The *“Omics*” Science comprises genomics, transcriptomics, proteomics and metabolomics technologies, which each provide valuable translational surveys in biological processes. A metabolomics approach enables the evaluation of metabolic pathways and the correlation of biochemical changes related to pathophysiology of disease, providing a downstream result of gene expression and higher sensitivity to phenotype of disease [40–45]. Underlying conditions and factors related to the occurrence, severity or prognosis of diseases with complex determinants may be assessed, bringing to light the final product of organism metabolism: the metabolome [42].

The development of a two-phase metabolomics research program that includes two large cohorts of nulliparous women is not an easy task. The network collaboration is essential to develop, implement and analyse such complex data and, more importantly, to achieve reliable results. Precise protocols for sample collection, processing, storage and biobank management will be essential to assure high quality data and results.

Metabolomics profiling requires different techniques to address the detection and quantification of different classes of metabolites once there is no current method capable to identify all of them. Preterm SAMBA study will employ different untargeted techniques that require very carefully and standardized protocols for sample preparation [24]. Studying the metabolome in blood samples requires invasive collection and immediate processing. As an alternative, hair samples are non-invasive, do not need processing methods and can be stored at room temperature. Hair can, theoretically, reflect endogenous compounds and environmental exposures from many days/weeks ago. The determination of the hair metabolome is a possible approach to identify biomarkers for spontaneous preterm birth. It has already been explored in gestational diabetes and fetal growth restriction, revealing potential endogenous mechanisms involved in those pathologic conditions [36, 45].

The identification of spontaneous preterm birth predictors using multi-ethnic data/samples and the evaluation of performance in a culturally and ethnically different population is desirable and meaningful for external validation. The use of quality control records and SPREC protocol is another important recommendation for metabolomics studies due to the necessity to evaluate confounders for analytical measures such as the time between sample collection, storage and processing conditions and the occurrence of haemolysis, lipaemia and metabolic degradation on account of inadequate temperature or solar exposure [37].

In the context of translational research, metabolomics may enhance understanding of the underlying pathways, which lead to obstetric complications. Preterm SAMBA aims to identify and validate a predictive model for spontaneous preterm birth, but will also develop a biobank and database that will enable research on pre-eclampsia (PE), fetal growth restriction (FGR) and gestational diabetes mellitus (GDM). The possibility to combine biochemical, genetics and clinical information that can be large-scale and replicable empowers the development of knowledge for clinical practice in preterm birth prevention. This would be especially worthwhile and helpful for countries with a high proportion and high absolute number of preterm births as is the case of Brazil where around 12 % of all births occur prematurely [46].

A recent clustered designed study showed that 30 % of all spontaneous preterm births do not have any maternal, fetal or placental conditions identified that could be related to its occurrence [47]. The application of metabolomics techniques could be a promising approach for spontaneous preterm birth prediction, all the more in those cases of silent phenotype in which there are no known predictors. Metabolomics have been already described in other obstetric conditions as pre-eclampsia, gestational diabetes mellitus and fetal growth restriction [31, 40, 41, 45, 48, 49], showing excellent performance in terms of a discriminatory algorithm. Therefore, we believe metabolomics is a powerful and strategic key not only for preterm birth prediction, but hopefully also for its prevention. The detection of metabolic pathways related to PTB syndrome may enable the development of more accurate therapies for primary or secondary prevention of pregnant women identified as at high-risk.

At the end of the study, if we are successful in the identification of such an effective algorithm, certainly several other topics should be carefully considered. Can this knowledge be really translated into a commercially available kit for screening purposes? Would the costs derived from this process be acceptable for low and middle-income countries? How will this be made available for populations in public sector? For discussing a future implementation of such a screening strategy, the following necessary points to be covered are to know if a concrete package of interventions to reduce preterm birth among those women identified as high-risk is available, and if it is cost-effective to be supported by the public health system. Finally, in this study we are planning to transfer the technology developed for the algorithm from New Zealand to Brazil, including lab technologies for assessing the biomarkers identified by metabolomics for preterm birth. Hopefully, if this is proved to be feasible, we believe that an important step for reducing the burden of preterm birth will have been achieved.

## Abbreviations

ADA, American Diabetes Association; BMI, body mass index; CONEP, Brazilian National Committee for Ethics in Research; FAME, fatty acid methyl esterification; FDA, Food and Drug Administration; FGR, fetal growth restriction; GC, gas chromatography; GDM, gestational diabetes mellitus; LC, liquid chromatography; MS, mass spectrometry; PE, pre-eclampsia; PI, principal investigator; pPROM, preterm premature rupture of membranes; PTB, preterm birth; SAMBA, Screening and Metabolomics in Brazil and Auckland; SCOPE, Screening for Pregnancy Endpoints study; SOP, standard operation procedures; sPTB, spontaneous preterm birth; WHO, World Health Organization
